# Additional abstracts, *JPGN reports, volume 5, issue S1*

**DOI:** 10.1002/jpr3.12100

**Published:** 2024-07-05

Due to a technical error, 24 abstracts that should have been published as part of the ESPGHAN 56th Annual Meeting Abstracts (JPGN Reports, volume 5, issue S1; doi:10.1002/jpr312073), published on 14 May 2024, were omitted.

Following publication, the omission was identified, and we have now corrected the error by adding the missing abstracts to the published issue on 05 July 2024.

ESPGHAN and Wiley apologize for this error.

The details of these abstracts are included below.

Kadirkhodjaeva K, Inoyatova F, Zakhidjanovna G, et al. The significance of allelic mutations of the HFE gene during chronic hepatitis b in children. JPGN Rep 2024;5(S1). doi:10.1002/jpr312073

Sokal E, Miethke A, Moukarzel A, et al. Maralixibat leads to improvements in cholestatic pruritus for children with progressive familial intrahepatic cholestatsis due to MDR3 deficiency: data from the march/march‐on trials. JPGN Rep 2024;5(S1). doi:10.1002/jpr312073

Yedvab M, Heiman E, Orlanski‐Meyer E, et al. Incidence and outcome of elevated alanine aminotransferase in infants with failure to thrive. JPGN Rep 2024;5(S1). doi:10.1002/jpr312073

Beime J, Schulz‐Juergensen S, Schild R, et al. Sustained response to IBAT treatment for severe pruritus in two children with nephronophtisis. JPGN Rep 2024;5(S1). doi:10.1002/jpr312073

Nassar R, Ealiwa N, Hassan L, et al. The clinical course of Wilson disease among children in southern Israel. JPGN Rep 2024;5(S1). doi:10.1002/jpr312073

D'Antiga L, Miethke A, Mogul D, et al. Maralixibat leads to significant reductions in bilirubin for patients with progressive familial intrahepatic cholestasis: data from the march trial. JPGN Rep 2024;5(S1). doi:10.1002/jpr312073

Pinon M, Vandriel S, Ng V, et al. Natural variation of liver transaminases in children with alagille syndrome: implications for evaluating drug‐induced liver injury with novel therapies. JPGN Rep 2024;5(S1). doi:10.1002/jpr312073

Vandriel S, Loomes K. Piccoli D, et al. Surgical interruption of the enterohepatic circulation is associated with an increased risk of liver transplantation or death in children with alagille syndrome. JPGN Rep 2024;5(S1). doi:10.1002/jpr312073

Chiadò C, Pinon M, Giugliano L, et al. Immunosuppressive therapy with everolimus in paediatric liver transplant: a single‐centre experience. JPGN Rep 2024;5(S1). doi:10.1002/jpr312073

Laue T, Ohlendorf J, Mutschler F, et al. Intensified immunosuppression with MMF is a risk factor for non‐immunity after hepatitis a vaccination ‐ a single‐centre, retrospective, observational analysis in paediatric liver transplant patients. JPGN Rep 2024;5(S1). doi:10.1002/jpr312073

Junge N, Di Giorgio A, Taibi L, et al. Quality of life in children with Crigler‐Najjar syndrome. JPGN Rep 2024;5(S1). doi:10.1002/jpr312073

Seisenbacher J, Kavallar A, Mayerhofer C, et al. Efficacy of percutaneous transhepatic cholangiography and drainage for biliary strictures after pediatric liver transplantation – a systematic review and meta‐analysis. JPGN Rep 2024;5(S1). doi:10.1002/jpr312073

Yankouskaya K, Allabauer I, Woelfle J, et al. Clec9a expression on Kupffer cells is downregulated in disease livers. JPGN Rep 2024;5(S1). doi:10.1002/jpr312073

Cucinotta U, Dipasquale V, Mayer C, et al. Does the scope (sclerosing cholangitis outcomes in pediatrics) index effectively predict later liver transplantation in children with sclerosing cholangitis? JPGN Rep 2024;5(S1). doi:10.1002/jpr312073

Drab A, Jańczyk W, Naorniakowska M, et al. Wilson disease diagnostic score evaluation in a single center pediatric study. JPGN Rep 2024;5(S1). doi:10.1002/jpr312073

Kumar K, Kaur J, Malhotra S, et al. Whole exome sequencing in pediatric acute liver failure – a single center experience. JPGN Rep 2024;5(S1). doi:10.1002/jpr312073

Friedman J, Nash M, Wesolowski S, et al. Alterations in liver and immune cell microenvironments in juvenile nonhuman primate offspring exposed to maternal western style diet as revealed by single cell RNA‐seq analysis. JPGN Rep 2024;5(S1). doi:10.1002/jpr312073

Martin Masot R, Navas‐López V, Santamaría Orleans A, et al. Use of comiss tool by Spanish paediatricians: does it really help with CMPA diagnosis? JPGN Rep 2024;5(S1). doi:10.1002/jpr312073

Hammond B. Lutein and zeaxanthin supplementation confirms cognitive performance benefits in children. JPGN Rep 2024;5(S1). doi:10.1002/jpr312073

Nazmi F, Yuksel M, Khan S, et al. Multiparametric Immunological analysis unveils aberrant regulatory t‐cell homeostasis in autoimmune hepatitis compared to sars‐cov‐2 hepatitis in children. JPGN Rep 2024;5(S1). doi:10.1002/jpr312073

Jugulete G, Borcos B, Safta M, et al. Efficacy and tolerability of entecavir in the treatment of chronic hbv hepatitis in children. JPGN Rep 2024;5(S1). doi:10.1002/jpr312073

Wierzbicka‐Rucińska A, Jańczyk W, Kułaga Z, et al. Hyaluronic acid in children with simple obesity and obesity combined with nafld or primary hypertension (hp). JPGN Rep 2024;5(S1). doi:10.1002/jpr312073

Lima R, Nau A. Factors associated with mortality from schistosomiasis: a populational study. JPGN Rep 2024;5(S1). doi:10.1002/jpr312073

Kaur P, Khanna R, Sood V, et al. Sarcopenia affects survival with native liver in children with chronic liver disease. JPGN Rep 2024;5(S1). doi:10.1002/jpr312073

